# Tuberculosis of the Femur With Intramedullary Abscesses and No Pulmonary Foci in an Immunocompetent Male: A Rare Case

**DOI:** 10.7759/cureus.61928

**Published:** 2024-06-07

**Authors:** Sankalp Yadav, Gautam Rawal, Naveen Jeyaraman, Madhan Jeyaraman

**Affiliations:** 1 Medicine, Shri Madan Lal Khurana Chest Clinic, New Delhi, IND; 2 Respiratory Intensive Care, Max Super Speciality Hospital, New Delhi, IND; 3 Orthopaedics, ACS Medical College and Hospital, Dr. MGR Educational and Research Institute, Chennai, IND; 4 Clinical Research, Viriginia Tech India, Dr. MGR Educational and Research Institute, Chennai, IND

**Keywords:** intramedullary abscesses, mycobacterium tuberculosis (mtb), cartridge based nucleic acid amplification test (cbnaat), tuberculosis, femur tb

## Abstract

Tuberculosis of the long bones/femur, especially in an immunocompetent person, is a challenging diagnosis. It is a rare entity, even in endemic settings. The non-specific clinical features, backed by a low suspicion about such presentations even in endemic settings, may result in delayed diagnosis and often unfavorable treatment outcomes. The situation becomes even more challenging in the absence of pulmonary foci and a contact history of tuberculosis. Here is a case of a young adult male who presented with complaints of pain over his left leg for three months. A diagnosis was achieved with magnetic resonance imaging and the isolation of the bacteria from a bone biopsy using a cartridge-based nucleic acid amplification test. Antituberculous treatment was promptly initiated.

## Introduction

Tuberculosis is a significant health issue for the general population in endemic settings [1]. One of the oldest known diseases to mankind, tuberculosis stands only second to COVID-19 in terms of mortality. It is presumed that it will claim its top spot in the coming post-severe acute respiratory syndrome coronavirus 2 (SARS-CoV) pandemic years [2]. In India, extrapulmonary tuberculosis constitutes about 15-53% of the total cases of *Mycobacterium tuberculosis* infection [3]. The majority are localized to the lymph nodes, pleura, and spine [4]. However, tuberculosis of the long bones is also documented. Although infrequently reported, bones like the femur can be infected after direct or indirect transmission [5].

Tuberculosis of the bones constitutes 25% of all extrapulmonary tuberculosis and is usually present in the spinal column, hip joint, and greater trochanter of the femur [6,7]. The incidence of involvement of the diaphysis of long bones in skeletal tuberculosis is extremely low, ranging from 1% to 3% in both endemic and non-endemic countries [8]. A case of a 19-year-old male is presented here, who was diagnosed with tuberculosis of the femur after a detailed workup.

## Case presentation

A 19-year-old Indian male from a low socioeconomic background presented with complaints of pain over his left knee. He had this pain for the past 1.5 years after a fall from his bicycle, but an exacerbation over the past three months resulted in his visit to the outpatient department. There were no other constitutional symptoms of tuberculosis. Also, there was no history of tuberculosis or cancer in the family or contacts. He was a student with no illicit drug history.

A general examination was suggestive of a hemodynamically stable male with an ectomorphic build. There was no clubbing, pallor, cyanosis, koilonychia, pedal edema, or palpable lymph nodes. A local examination was suggestive of tenderness over the distal part of the anterior left leg. The active and passive range of motion of the knee was 0-60 degrees and painful beyond it. However, there was no visible swelling, scar, or discharging sinus. His systemic examination was unremarkable.

**Figure 1:**
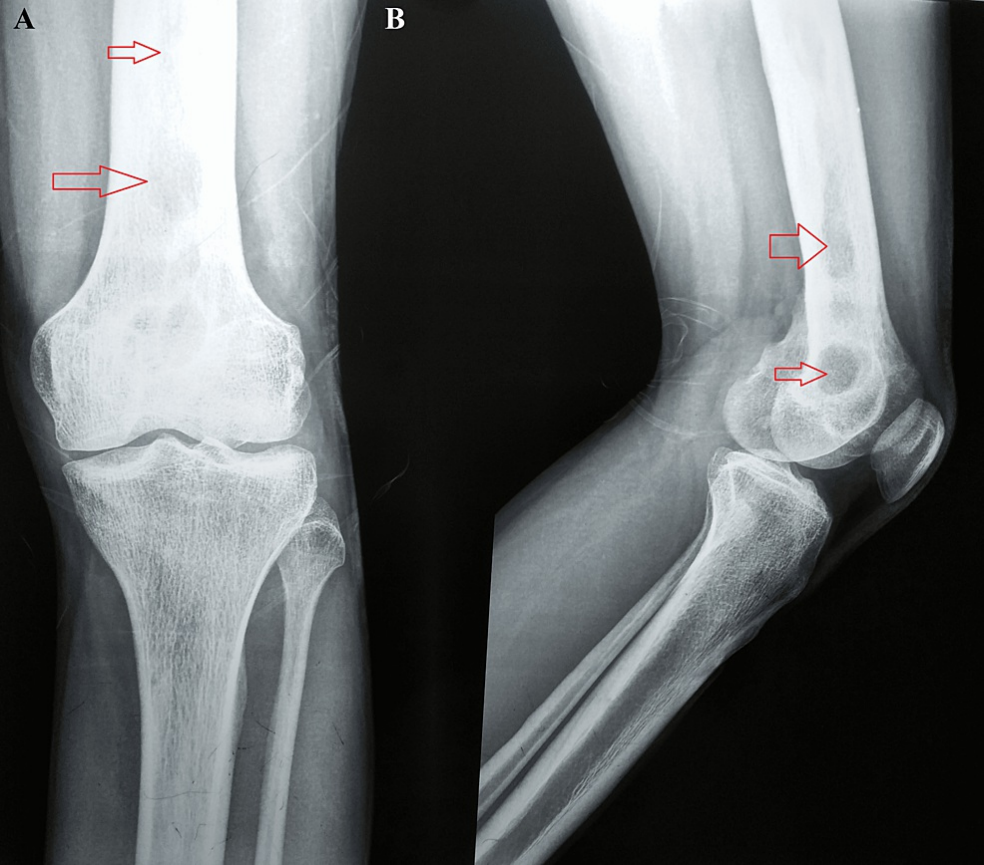
A plain radiograph of the knee suggestive of multiple osteolytic lesions in the distal metadiaphyseal region of the left femur A: anteroposterior view; B: lateral view

**Figure 2:**
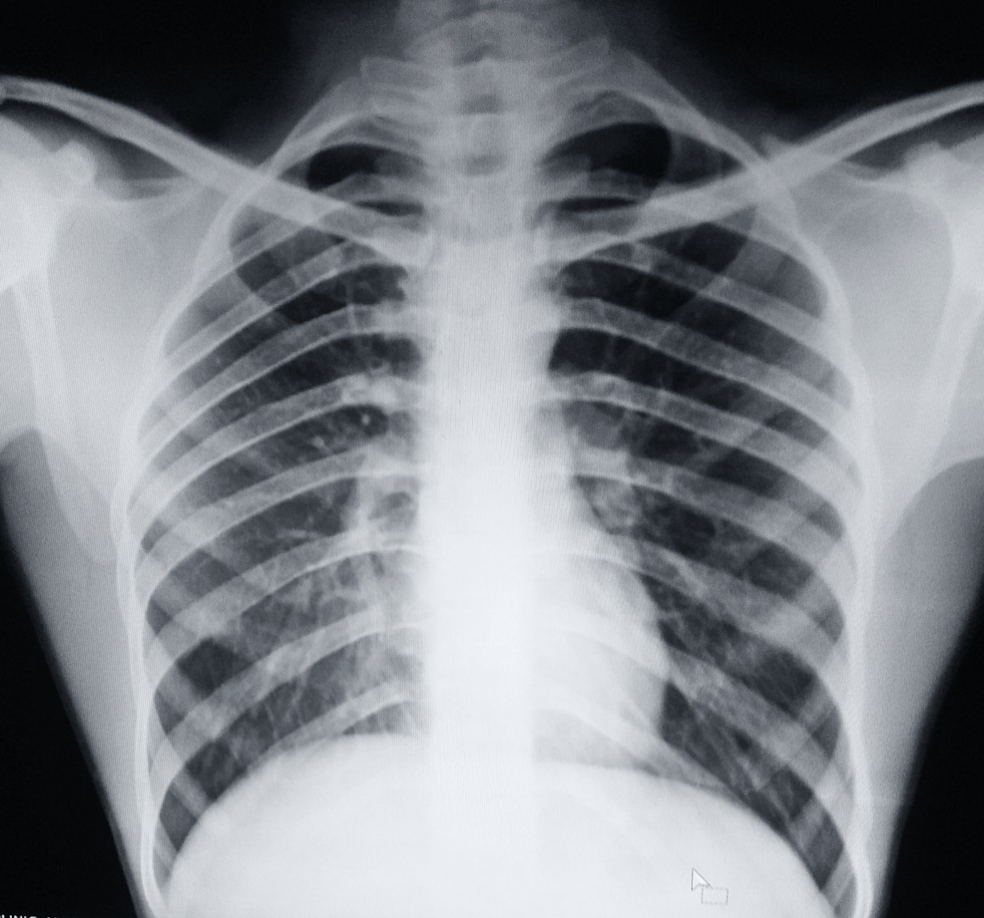
Chest radiograph was normal (posteroanterior view)

A plain radiograph of the knee was suggestive of multiple osteolytic lesions in the distal metadiaphyseal region of the left femur (Figure 1). A chest radiograph was taken but it was normal (Figure 2).

Blood panel was within the reference range except for a raised erythrocyte sedimentation rate (40 mm/hr) and C-reactive proteins (45.20 mg/L). He was non-diabetic, and his HIV (I and II) and hepatitis (A, B, and C) tests were negative. His Mantoux test was positive with 20 mm of induration. Further, an induced sputum microscopy and cartridge-based nucleic acid amplification test (NAAT) were negative.

**Figure 3:**
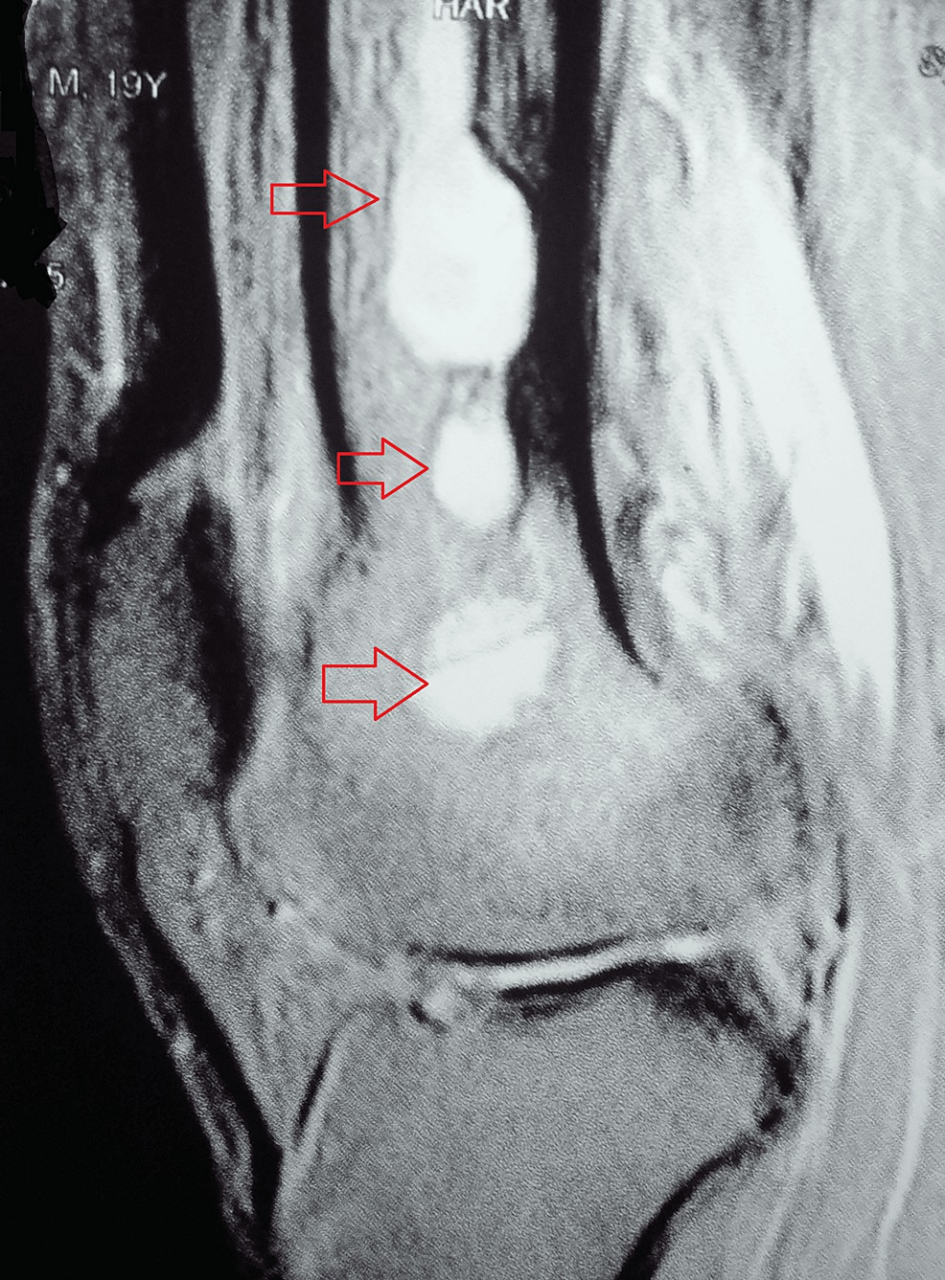
MRI of the left knee suggestive of multiple small intramedullary areas of fluid collection with diffuse perifocal edema involving the femoral shaft and supracondylar region MRI: magnetic resonance imaging

Magnetic resonance imaging of the left knee was suggestive of multiple small intramedullary areas of fluid collection, appearing hyperintense on T2/short tau inversion recovery (STIR) images and isointense on T1W images with diffuse perifocal edema involving the femoral shaft and supracondylar region (Figure 3).

Due to the endemicity of tuberculosis, a provisional diagnosis of tuberculosis of the femur with differentials such as cystic bone lesions, bone tumors, Brodie's abscess, persistent pyogenic osteomyelitis, or fungal or bacterial granulomatous lesions was made.

To confirm the diagnosis, the patient went through a percutaneous biopsy of the left femur guided by a computed tomography scan. The cultures of the bone biopsies revealed no signs of fungus or aerobic and anaerobic organisms. Additionally, acid-fast bacilli smears were negative. There was no evidence of active osteomyelitis found in the histopathology of the bone biopsy. However, a sample sent for a cartridge-based NAAT detected low *M. tuberculosis* with no resistance to rifampicin. Hence, a final diagnosis of tuberculosis of the femur with intramedullary abscesses and no pulmonary foci was made, and he was initiated on antituberculous treatment for 12 months (two months of intensive phase with four drugs, i.e., rifampicin, isoniazid, pyrazinamide, and ethambutol, and 10 months of continuation phase with three drugs, i.e., rifampicin, isoniazid, and ethambutol). Additionally, tablet diclofenac 50 mg was added for pain, and tablet ranitidine 150 mg was added twice daily.

He was counseled for treatment adherence, maintaining hygiene, and strict non-weight bearing on his left leg. Also, liver function tests were done monthly, which were normal. He fared well in the first two months with no major adverse drug reactions; at four weeks of the intensive phase, static quadriceps strengthening exercise, knee bending exercise, and non-weight-bearing on the left leg walking with walker support were advised under a physiotherapist. At six weeks of treatment initiation, he progressed to partial weight-bearing walking. He continued his treatment for the next four months and then left the district for his village. He continued his treatment there for a total of 12 months with significant improvement and was declared cured at 12 months in the National Tuberculosis Elimination Programme data portal, Ni-kshay [9].

## Discussion

Skeletal tuberculosis makes up 1-2% of total tuberculosis cases and 10% of extrapulmonary cases [4]. Long-bone skeletal tuberculosis is a prime example of a medical imitator [8]. The non-specific presentation of tuberculosis in long bones, with generalized pain and swelling and minimal symptoms of inflammation, makes it a persistent challenge even with advances in medicine [10]. Even though the respiratory system is the main point of entry, it continues to be mostly dormant in skeletal tuberculosis. Per the hypothesis, tubercular osteomyelitis typically arises from the initial lung infection's lymphohematogenous spread to the bone, followed by local reactivation. The most frequent location of the primary lesion is the metaphysis, from which it may either progress to the adjacent joint in the epiphysis or remain harbored [11]. Diaphysis involvement is infrequent [8].

Due to similarities with more common differentials such as cystic bone lesions, bone tumors, Brodie's abscess, persistent pyogenic osteomyelitis, or fungal/bacterial granulomatous lesions, tuberculosis in long bones, which typically presents as a single lesion with no specific signs and symptoms, often goes unnoticed [8]. The average delay in diagnosing bone tuberculosis was reported to be 6.6 months in a study by Akgul et al. [12].

The infrequency of tubular disease incidence in bones and the lack of a recognizable radiological marker further contribute to the neglect of a proper diagnosis [8,13]. To make a final diagnosis for such a condition, clinicians must combine their understanding of the potential differentials with radiological examinations, evaluate the findings, and combine that with histopathological and culture sensitivity data [10]. Management is essentially conservative after drainage of the pus with antituberculous drugs for 12 months [4]. In early cases, surgery is rarely recommended. Only in cases with severe disease or when medical therapy is not improving the condition is radical debridement suggested. Individuals who develop severe joint involvement may require total joint arthroplasty or arthrodesis [14].

A case similar to the present case was reported by Rajavel et al. [11]. However, unlike their case, there were multiple abscesses in the intramedullary region of the left femur in the present case. Moreover, a surgical excision biopsy and curettage were not done in their case and *M. tuberculosis *was isolated from the cartridge-based NAAT from a paucibacillary sample.

Despite a detailed literature search, we could not find another reported case of tuberculosis of the femur with intramedullary abscesses and without pulmonary involvement in an immunocompetent male. This stresses the need for reporting such rare presentations, which could be really helpful for the dissemination of knowledge about such infrequent presentations of a common disease.

## Conclusions

A case of tuberculosis of the femur with intramedullary abscesses without pulmonary involvement is a diagnostic challenge in an immunocompetent individual. The non-specific clinical features and ambiguity in the initial radiography could result in treatment failures. This case emphasizes the need for a high degree of clinical suspicion in cases like this in the outpatient department. Even in endemic settings, there is a scarcity of data and awareness among primary care clinicians about tuberculosis of the femur.
